# PrAna: an R package to calculate and visualize England NHS primary care prescribing data

**DOI:** 10.1186/s12911-021-01727-z

**Published:** 2022-01-06

**Authors:** Kishore Kumar Jagadeesan, James Grant, Sue Griffin, Ruth Barden, Barbara Kasprzyk-Hordern

**Affiliations:** 1 Department of Chemistry, University of Bath, Bath, UK; 2 Digital, Data and Technology Group, University of Bath, Bath, UK; 3 NHS Bath and North East Somerset Clinical Commissioning Group, Bath, UK; 4 Wessex Water, Bath, UK

**Keywords:** Prescription data, Health informatics, R, Pharmaceuticals, Prescription, R package

## Abstract

**Background:**

The objective of this work to calculate prescribed quantity of an active pharmaceutical ingredient (API) in prescription medications for human use, to facilitate research on the prediction of amount of API released to the environment and create an open-data tool to facilitate spatiotemporal and long-term prescription trends for wider usage.

**Design:**

We have developed an R package, PrAna to calculate the prescribed quantity (in kg) of an APIs by postcode using England’s national level prescription data provided by National Health Service, for the years 2015–2018. Datasets generated using PrAna can be visualized in a real-time interactive web-based tool, PrAnaViz to explore spatiotemporal and long-term trends. The visualisations can be customised by selecting month, year, API, and region.

**Results:**

PrAnaViz’s targeted API approach is demonstrated with the visualisation of prescribed quantities of 14 APIs in the Bath and North East Somerset (BANES) region during 2018. Once the APIs list is loaded, the back end retrieves relevant data and populates the graphs based on user-defined data features in real-time. These plots include the prescribed quantity of APIs over a year, by month, and individual API by month, general practice, postcode, and medicinal form. The non-targeted API approach is demonstrated with the visualisation of clarithromycin prescribed quantities at different postcodes in the BANES region.

**Conclusion:**

PrAna and PrAnaViz enables the analysis of spatio-temporal and long-term trends with prescribed quantities of different APIs by postcode. This can be used as a support tool for policymakers, academics and researchers in public healthcare, and environmental scientist to monitor different group of pharmaceuticals emitted to the environment and for prospective risk assessment of pharmaceuticals in the environment.

## Background

During the last decade a wide range of pharmaceuticals have been identified and quantified in the aquatic environment across several countries and their impacts on exposed environmental species [1–4] and humans [5] have been reported. However, currently it is not feasible to monitor all the pharmaceuticals released into the aquatic environment due to analytical feasibility and monetary limitations [6]. Recently, several procedures have been established to predict the total amount of pharmaceuticals emitted to the environment for risk assessment of pharmaceuticals in water by public authorities in both Europe [7, 8] and the United States [9]. These guidelines describe how to evaluate the risk of the pharmaceuticals in the environment, based on human pharmaceutical consumption, ecotoxicity of these pharmaceuticals [10] and their removal during wastewater treatment [11], where human pharmaceutical consumption plays a vital role [12].

In addition to its role in the prediction of environmental concentration in water, spatiotemporal data of human pharmaceutical consumption combined with other data could be used to explore and predict public health [13], for example, (1) prescription data for short-acting β2-agonists, and respiratory related prescribing could be used as a predictor for respiratory mortality and/or air pollution health effects [14–16], (2) antibiotic prescription data could be used as a predictor to measure antimicrobial resistance in the community [17–19], (3) reduction in the number of antidepressant prescriptions can be linked with an increase in the urban green space [20, 21], (4) opioids prescription monitoring could be used to assess community mental health [22, 23].

Despite its importance in environmental risk assessment and support in predicting public health, estimation of the human pharmaceutical consumption or the total manufactured amount is difficult. In those countries where the data is publicly available, it is complex and not straight-forward to handle. In the UK, since December 2011, the National Health Service (NHS) has made national prescription data publicly available at general practice (GP) level, reporting the item counts, quantity and cost of prescriptions aggregated by British National Formulary (BNF) code [13].

National prescription data from the NHS dataset typically contains over 10 million records per month. And this dataset cannot be used for the direct calculation of the consumption levels of different pharmaceutical drugs. We need to use BNF code and SNOMED code [24, 25] to identify the actual pharmaceutical ingredients and contents together with prescribed quantities to calculate the actual amount of each component dispensed. Open Prescribing provides some tools for data exploration for national level spatiotemporal trends for different pharmaceutical drugs using NHS prescription data [26]. However, these tools report used item counts for the drug volume, which has limitations [27], as it does not take the actual dosage or total amount of consumption of pharmaceuticals, which is crucial for the total amount of pharmaceuticals emitted to the environment.

In this article, we present PrAna, an R [28] package to aggregate and estimate the total prescribed quantity of active pharmaceutical ingredients (APIs) in prescription medications for human use in the UK. The PrAna package is complemented by a user-friendly interactive tool, PrAnaViz, developed using R/Shiny framework, where user can explore and visualise the spatiotemporal trends of the total prescribed quantity of APIs and can download the results in various data formats for their further studies.

## Methods

### Data sources

#### NHS prescription and practices location datasets

Monthly national level prescribing datasets for the years 2015 to 2018 were downloaded from NHS Digital [29]. Clinical Commissioning Groups (CCG) names and codes and CCG geographic boundaries, GP practices geographical location were downloaded from NHS Digital [30] and Office for National Statistics data portal [31] used under the terms of the Open Government Licence. Postcode information were downloaded from Office for National Statistics [32] licensed under the Open Government Licence v.3.0. NHS prescription datasets contain dispensed prescriptions from general practitioners and other non-medical prescribers (such as nurses, pharmacists, optometrists, chiropodists and potentially radiographers) but does not cover private prescriptions. Each dataset has more than 10,000 rows, with each row representing a prescription, containing information on the dispensed practice code, British National Formulary (BNF) code, BNF Name, number of items prescribed, net ingredient cost, actual cost, quantity, and period as in Table 1.

**Table 1:** NHS prescription dataset with description for each column

SHA	PCT	PRACTICE	BNF CODE	BNF NAME	ITEMS	NIC	ACT COST	QUANTITY	PERIOD
Q44	RTV	Y04937	0304010W0BBABAL	Phenergan_Tab 25 mg	3	8.15	7.89	98	201801
Q44	RTV	Y04937	0401010Z0AAAAAA	Zopiclone_Tab 7.5 mg	7	2.88	3.35	98	201801
Q44	RTV	Y04937	0401020K0AAAHAH	Diazepam_Tab 2 mg	5	3.76	3.94	191	201801
Q44	RTV	Y04937	0402010ADAAAAAA	Aripiprazole_Tab 10 mg	3	13.64	12.88	63	201801
Q44	RTV	Y04937	0402010ADAAADAD	Aripiprazole_Tab 5 mg	1	1.36	1.37	7	201801

Column	Description
SHA	From April 2013 onwards, this code refers to Area Team
PCT	From April 2013 onwards, this code refers to Clinical Commissioning Group (CCG) code
PRACTICE	GP Practice code
BNF CODE	Unique identifier to show what is prescribed
BNF NAME	Describes the formulation and strength as well as the drug’s brand or generic (product) name
ITEMS	Number of prescription items dispensed
NIC	The drug tariff price in pounds and pence, which may be subject to further charges and/or discounts
ACT COST	Actual cost—pounds and pence
QUANTITY	Quantity represents the quantity of a drug dispensed, with units of measurement (units/tablets/grams/millilitres, etc.) dependent on its formulation
PERIOD	Prescribed year and month

As we are interested in calculating the total quantity of an individual active pharmaceutical ingredient (API), the whole dataset needs to be evaluated for each API. The columns BNF code and BNF name could help us in the normalisation, as they carry the information regarding the API. The BNF code is a 15-digit code unique for each formulation, dose and product combination. Together with the quantity prescribed, we use this to identify the amount of each API dispensed with each prescription.'

#### BNF/SNOMED mapping data

The BNF code in the 2015 to 2018 annual NHS prescription datasets uses the legacy Master Data Replacement (MDR) drug database [29]. We map these BNF codes to individual API with BNF/SNOMED mapping data (Table 2) provided by the NHS Business Services Authority (BSA) on June 2018 [24], BNF/SNOMED mapping data map legacy MDR drug database and the Dictionary of Medicines and Devices (dm + d) [33]. Once we have mapped each BNF in the dataset to its APIs we can evaluate the quantities prescribed in the NHS prescription dataset.

**Table 2:** BNF SNOMED mapping—June 2018

BNF code	VMPP/AMPP SNOMED code	MDR: product description
'0501050B0BEAAAE	'18149311000001103	Clarie XL_Tab 500 mg
'0501050B0BEAAAE	'18149411000001105	Clarie XL_Tab 500 mg
'0501050B0AAACAC	'34751711000001107	Clarithromycin_I/V Inf 500 mg Vl
'0501050B0AAACAC	'13469811000001104	Clarithromycin_I/V Inf 500 mg Vl
'0501050B0AAACAC	'17997811000001100	Clarithromycin_I/V Inf 500 mg Vl
'0501050B0AAACAC	'13613011000001105	Clarithromycin_I/V Inf 500 mg Vl

#### Data aggregation

First step to quantify an individual API from NHS prescription dataset is to map ‘VMPP/AMPP SNOMED’ code in the BNF SNOMED mapping dataset to an individual chemical substance. To achieve this, ‘VMPP/AMPP SNOMED’ code in the BNF SNOMED mapping dataset was matched to the Actual Medicinal Product (AMP) and those without matching AMP were matched to Virtual Medicinal Product (VMP) with help of dm + d files. In some cases, ‘VMPP/AMPP SNOMED’ code was matched to Virtual Medicinal Product Pack (VMPP) or Actual Medicinal Product Pack (AMPP). Once the ‘VMPP/AMPP SNOMED’ codes were mapped to VMP/AMP or VMPP/AMPP, the later helps to match the former to individual chemical substance. This mapping also enables to identify the medicinal form (e.g., tablet, capsule, solution for injection, etc.), strength with its unit of measurement (e.g., mg, ng, µg, etc.).

For example, consider amoxicillin 500 mg capsules, with our method we have managed to find 50 unique ‘VMPP/AMPP SNOMED’ codes for this and it is matching to 1 unique BNF code, 4 VMPPs, 28 AMPs with 52 AMPPs. For this example, we have identified API as amoxicillin (as amoxicillin trihydrate), medicinal form as capsules, strength as 500 mg and four different pack levels 15 capsules, 28 capsules, 100 capsules and 21 capsules respectively, from 28 different manufacturers.

Our method also helps to differentiate API from the formulations containing more than one API, for example, consider co-amoxiclav 500 mg/125 mg tablets, with our method we have managed to find 24 unique ‘VMPP/AMPP SNOMED’ codes for this and it is matching to 1 unique BNF code, 2 VMPPs, 23 AMPs with 24 AMPPs. For this example, we have identified APIs as amoxicillin (as amoxicillin trihydrate) and clavulanic acid (as potassium clavulanate) with strength 500 mg and 125 mg respectively, medicinal form as tablet, and two different pack levels 21 tablets and 100 cap tablets respectively, from 26 different manufacturers.

In the second step, the ‘BNF code’ from the dataset generated as above, was matched to the BNF code in the NHS prescription dataset. This enables to identify individual API, along with information on its medicinal form, strength, and its unit of measurement for each row in the NHS prescription dataset.

**Fig. 1:**
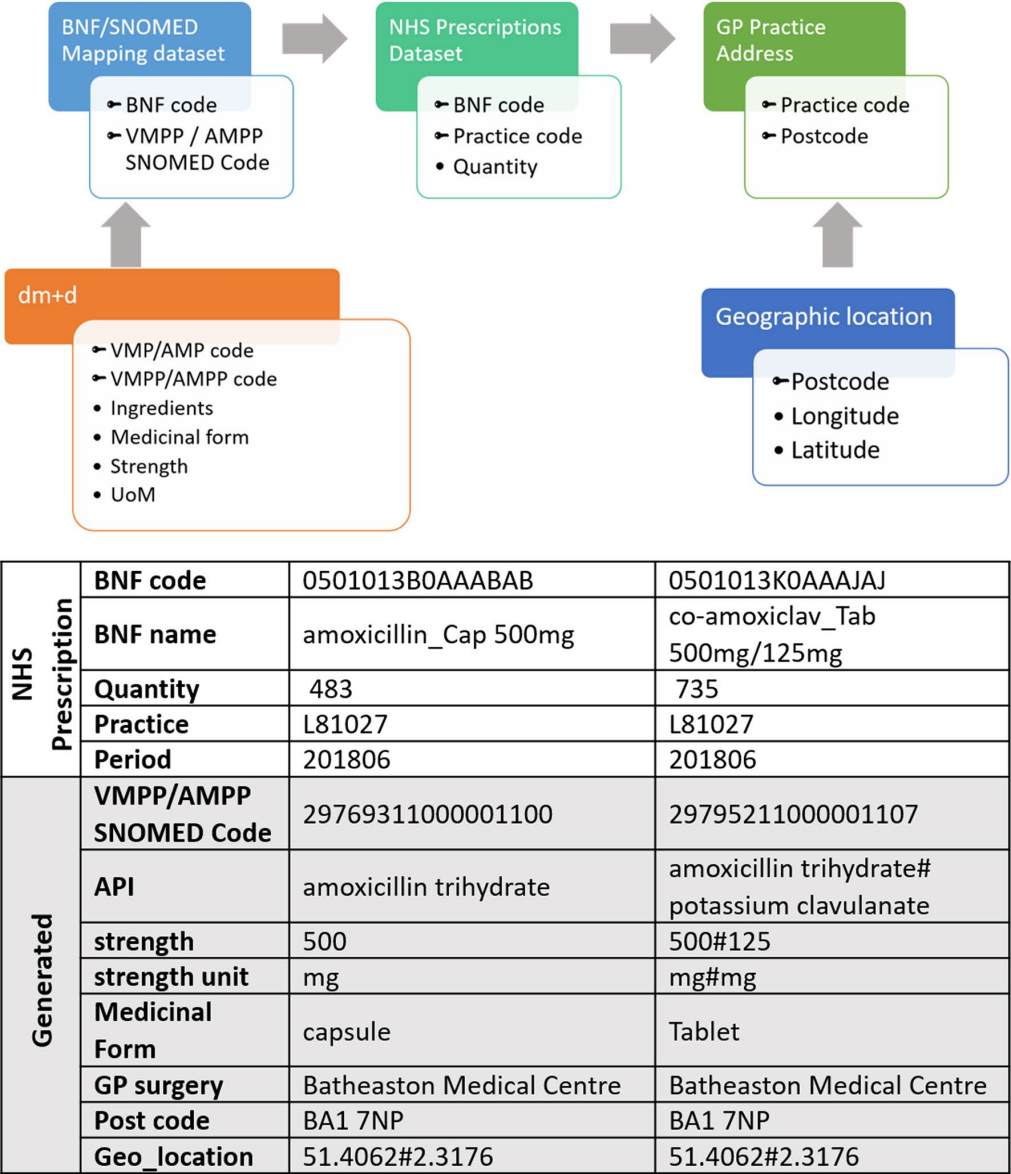
Data aggregation: Data aggregation and normalisation flow chart describing how API, strength, medicinal form, GP surgery name were matched to the BNF code in the NHS Prescription dataset. Examples of the matched API are given in the table. The first example shows the BNF code matching to one API and prescribed as a capsule, and second one shows the matching to two different APIs and prescribed as a tablet

After the identification of API and its strength, the dataset is grouped by individual API, by GP practice code and ultimately by postcode and the total prescription quantity for individual API was calculated for each GP practice code and postcode. We have used ‘Quantity’ from the NHS prescription dataset to measure total prescription quantity. Postcode and geographical coordinates were linked to the dataset by matching the GP practice code from the dataset downloaded from NHS Digital and Office for National Statistics data portal. The whole process is summarised below in Fig. 1. Full methodology for this complete matching process is available in the technical documentation online [34].

### PrAna: an R package implementation

PrAna is an R package providing a comprehensive workflow including data preparation, data conversion, data visualisation and the ability to export/download the generated plots and data, as outlined in the Table 3. The package is now available from https://github.com/PrAnaViz/PrAna under an MIT license and we are in the process of uploading it in the Comprehensive R Archive Network (CRAN). Deploying the PrAna requires R version 3.5 or greater and a small number of package dependencies, detailed instructions can be found in the online documentation [34].

**Table 3:** PrAna workflow

Workflow	Description
Data preparation	Download monthly NHS prescription datasets and Dictionary of medicines and devices release files (dm + d)
Data conversion	Aggregation and conversion of the locally stored datasets into practice wise dataset achieved using the functions in PrAna
Data visualisation	Visualise and analyse the processed dataset using the in-built ShinyApp PrAnaViz
Download	Download processed data as.csv file and publication ready image.eps and.pdf files

#### PrAna installation

Several functions wrapped in the PrAna package utilised in the workflow, we recommend using RStudio to install PrAna, to keep directories specific to a single *`.Rproj*`. Since the code is published on GitHub, it can be installed using *`devtools`*. With *`devtools`* installed you can download and install the latest version of *'PrAna'* in the R console with *`install_github("PrAnaViz/PrAna”)`*.

#### Data preparation

It is strongly suggested to setup the destination folder as the working directory using *setwd()* function. `*csv2dat()`* function is used to combine several monthly NHS prescription dataset “Practice Level Prescribing Presentation-level Data: Practice prescribing data” files, downloaded from NHS digital [29] into a single data table. For example, if the user wants to combine all the monthly prescription dataset downloaded in the *"C:/Datasets/Prescription Datasets/2018/PDPI"* location to a destination folder “*C:/Datasets/2018”*, following commands need to be executed in the R console.
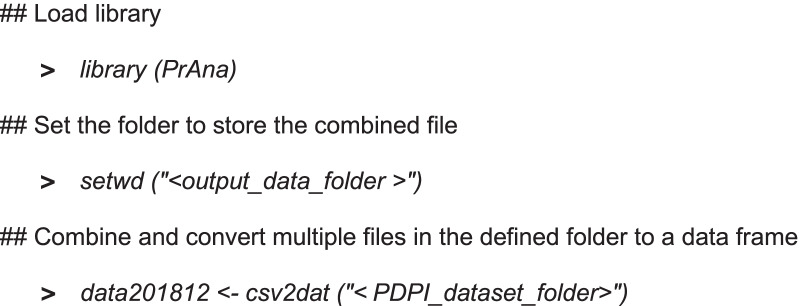


*`importdmd()`* function is used to import different extracted dm + d files [33] and return multiple data objects including a data table which map each BNF code to its corresponding API(s), strength and medicinal form. Recommended to read the documentation of *importdmd()* function to know more regarding the different data objects it generate [34].

## Read the extracted dm + d files
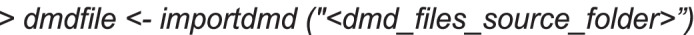


To identify and generate GP practices list for each CCG, “Practice Level Prescribing Presentation-level Data: Practice codes, names and addresses” (ADDR) file from NHS Digital [30], CCG Generalised Clipped Boundaries in England shape files [35] and National Statistics Postcode Lookup (NSPL) files [32] from ONS will be utilised, and functions `*combine_addr()`* and `*generate_gp_list_ccg()`* are used.

First step, is to use the function `*combine_addr()`* to generate a combined ADDR file.
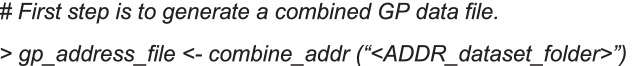


The file will have columns: GP practice code, name, and address with postcode.

The next step is to generate the list of GP practices for the defined CCG region using the function *`generate_gp_list_ccg()`.*

This function requires following parameters,

- *shape_file*—Generalised Clipped Boundaries of CCG regions in England [35]
- *postcode_ons*—NSPL file [32]
- *gp_data_file*—*gp_address_file* generated in the using *`combine_addr()`* function
- *region*—Name of the CCG region

Briefly, this function identifies the postcodes of the GP practices from the ADDR files and finds its geographic location from NSPL file by matching the postcodes. To estimate the GP practices within each CCG region, generated GP practices geographic locations were intersected with the freely available geospatial shape file of high resolution CCG maps [35].
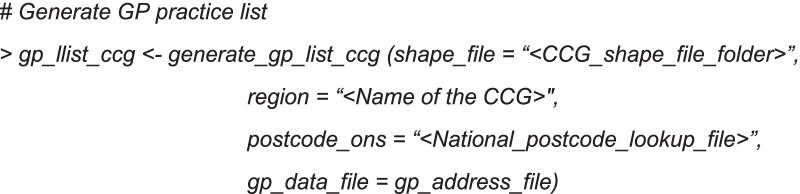


#### Data normalisation and conversion

The final step in the data conversion is to use *practice_wise()* to generate the prescription dataset mapped with the individual API, prescription quantity, medicinal form, and strength for the defined GP practices. The practice_wise() function require following six parameters, as demonstrated in the example below,

- Combined NHS prescription dataset, generated using *csv2dat()* function.
- A character vector containing GP Practices.
- A data table containing BNF Code mapped to individual APIs, strength, medicinal form.
- Unit of measurement with multiplication factors file
- Different medicinal forms with its corresponding codes file
- Different APIs with its corresponding codes file

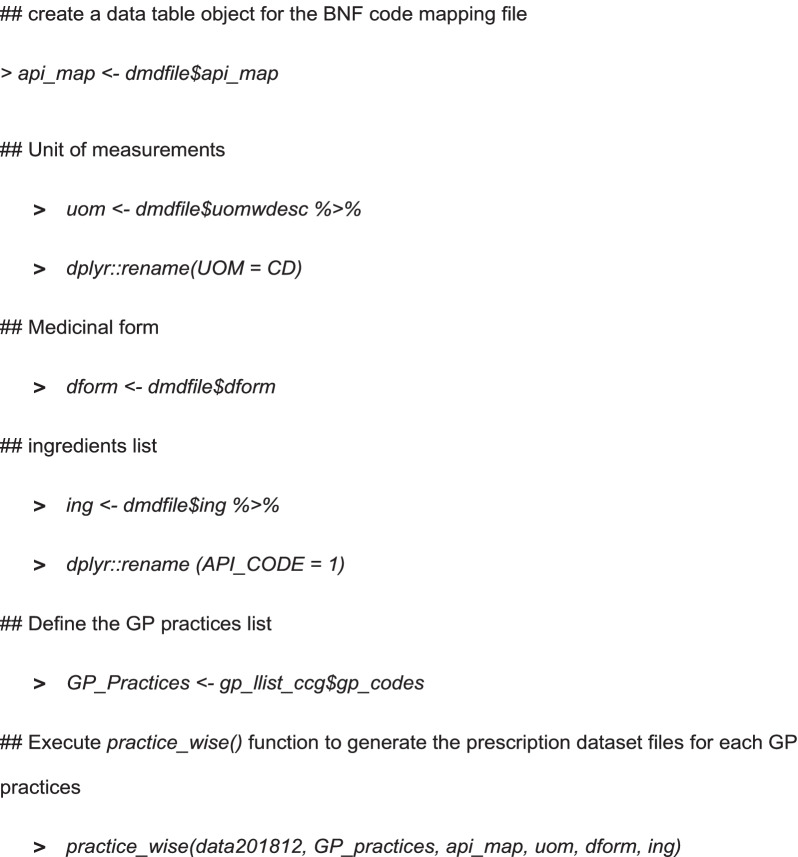


#### Database service

As a result of the large datasets, to run PrAnaViz we recommend uploading the processed dataset to a local or a remote database service, for example, MySQL, and link it to the PrAnaViz. More information on the linking databases to *PrAnaViz* is explained in our technical documentation online.

### PrAnaViz: an interactive data analysis tool

We developed an interactive tool PrAnaViz to visualise, explore and export different spatiotemporal prescription trends for wider use using the GP specific prescription data generated using PrAna package. Created using R/Shiny, PrAnaViz, uses a web-based interactive dashboard layout that most users are familiar with from common websites and web-based tools.

To launch PrAnaViz run the following command in your R Console:

-  > *library(PrAna)*
-  > *PrAna::runShiny("PrAnaViz")*

The *PrAna::runShiny("PrAnaViz")* function will pop-up the PrAnaViz tool which will allow you to explore different spatiotemporal and long-term prescription trends with the sample dataset.

The basis of PrAnaViz functionality is that any user can explore prescribing trends broken down by chemical substance, and to explore and visualise the variation in prescribing at region, postcode, and individual GP practice level, where both the overall trends and the relative contribution from each API, medicinal form can be seen. PrAnaViz contains two different dashboards tabs: (1) Targeted API approach in which a user can input their target(s), i.e., API(s) of interest and calculate, visualise, and explore total prescribed quantity of targeted API(s) in a selected region, (2) Non-targeted API approach where the user can select an individual API and visualise total prescribed quantity of that API at resolutions down to individual postcodes.

**Fig. 2:**
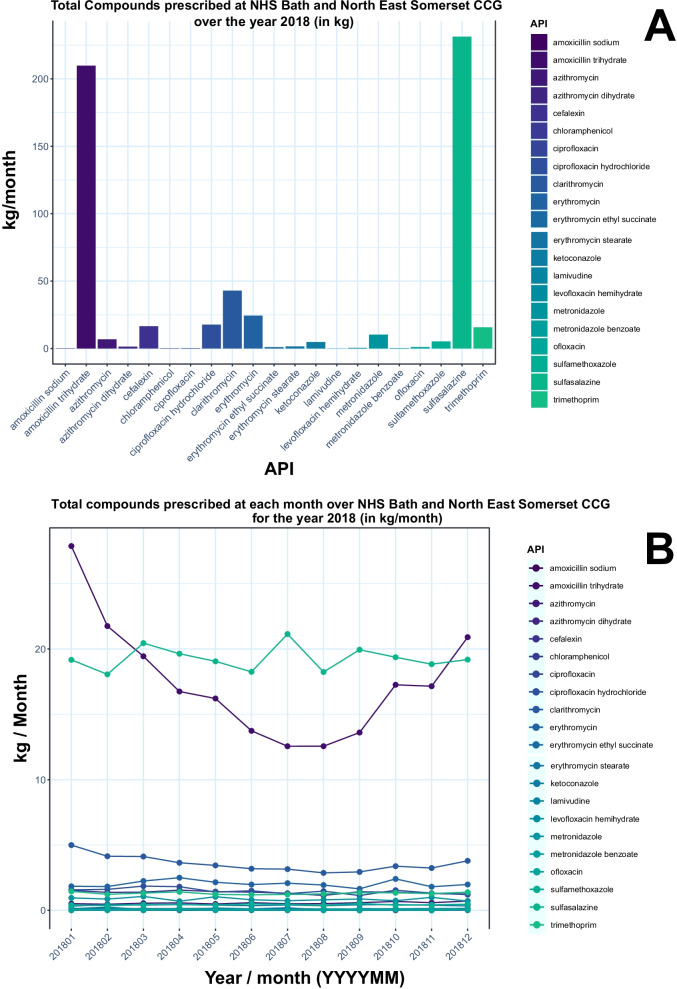
Visualising time series of prescription data (Targeted API approach): **A** Screenshot of graph showing prescription (in kg) of 14 APIs in Bath and North East Somerset CCG region in 2018. **B** Screenshot of graph showing monthly prescription (in kg) of 14 APIs at Bath and North East Somerset CCG in 2018

**Fig. 3:**
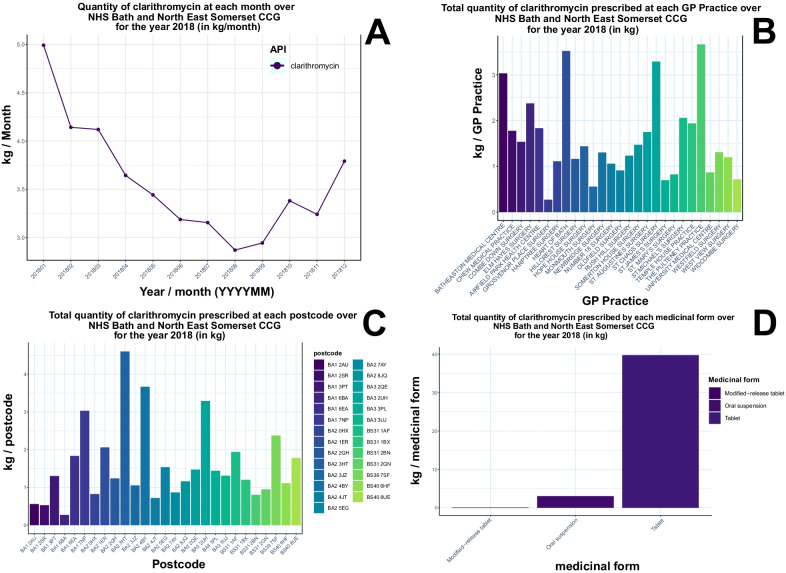
Exploration of different prescription data trends for an API target (targeted API approach): prescription quantity of clarithromycin by each month (**A**), by individual GP level (**B**), by individual postcode level (**C**) and medicinal form (**D**) for Bath and North East Somerset CCG in 2018. Figures are generated using PrAnaViz

**Fig. 4:**
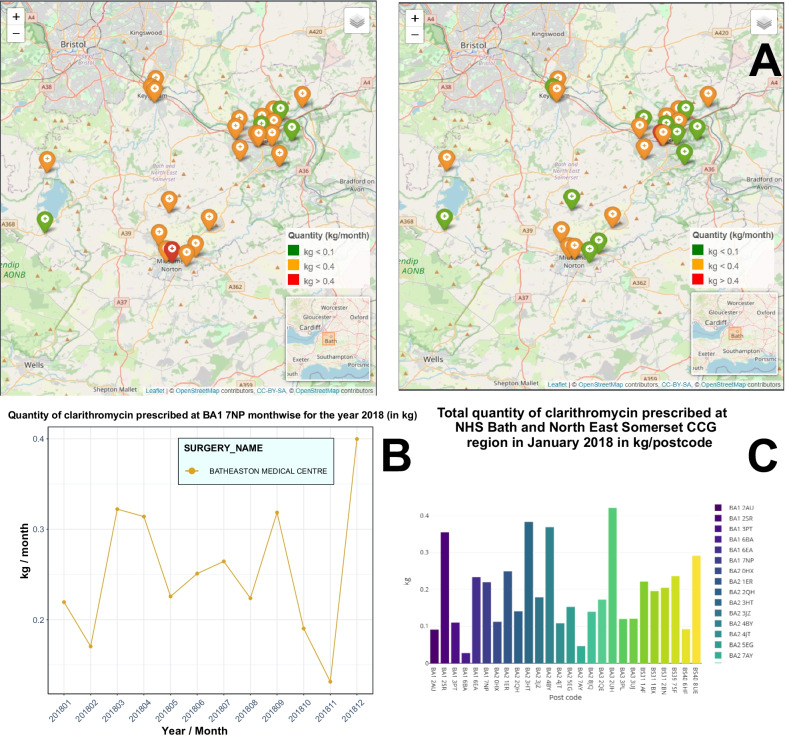
Visualising spatiotemporal series of prescription data (non-targeted API approach): **A** Total prescription quantity (in kg) of clarithromycin at the different postcodes in Bath and North East Somerset CCG region in January 2018 and June 2018. **B** Monthly prescription quantity (in kg) of clarithromycin at the postcode BA1 7NP at Bath and North East Somerset CCG region in 2018. **C** Pie-chart showing total prescription percentage of different medicinal forms of clarithromycin at the postcode BA1 7NP in Bath and North East Somerset CCG region in 2018. **D** Total prescription quantity (in kg) of clarithromycin at the different postcodes in Bath and North East Somerset CCG region in January 2018. Figures are generated using PrAnaViz. Base map tiles provided by OpenStreetMap © OpenStreetMap contributors. The digital boundary file contains Office for National statistics licensed under the Open Government Licence v.3.0, contains OS data © Crown copyright and database right (2018)

Users can input API target(s) of interest as a comma separated value (.csv) file, for the targeted API approach and can input the connection strings to connect their databases to the PrAnaViz. Detailed instructions on the input options are available in the supporting information and online tutorial documentation for PrAnaViz [34]. Users can export the graphs as a publication ready Encapsulated PostScript (.eps) file or Portable Network Graphics (.png) or Portable Document Format (.pdf) file and corresponding datasets as a.csv file according to selection criteria defined by the user to carry out their own analyses. The Figs. 2, 3 and 4 used in the article are generated using PrAnaViz.

## Results

### PrAna

We extracted the available monthly prescription dataset from January 2015 to December 2018 and mapped it to the prescribed general practices and by postcode. And the data generated will be in comma separate value (.csv) files dedicated to each general practice with file name corresponding to the GP code, we obtained the GP code from NHS Digital [30] and geographical coordinates to the postcodes from Office for National Statistics data portal [31]. This mapping also enables to identify the medicinal form (e.g., tablet, capsule, solution for injection, etc.), strength with its unit of measurement (e.g., mg, ng, µg, etc.), packing level information and manufacturer information. We then excluded compounds related to medical devices/appliances, bath additives, washes, foams, shampoos, sprays, multivitamin capsules, and tablets for our analysis.

### PrAnaViz

Information on prescribing quantity of different APIs in a particular year and a year-long trend of an API are interesting for various applications including community health monitoring [23, 36–38], environmental monitoring [39, 40], policy interventions [41], and environmental and water quality management [21, 42, 43]. This tool can facilitate research in these aspects and in the identification of most prescribed API in particular region or in a practice and able to identify the variation across the prescription of API over a period or region or practice. For example, Fig. 2A shows the annual prescription (in kg/year) of 15 APIs corresponding to antibiotics group in Bath and North East Somerset CCG region in 2018 and shows that amoxicillin (209.76 kg/year) and sulfasalazine (231.29 kg/year) are the most prescribed in the group at selected condition, but when comparing the same group of APIs in month wise prescription (in kg/month) Fig. 2B shows that like amoxicillin and clarithromycin, shows the higher prescription rate in winter comparing to summer, but sulfasalazine prescription is same throughout the year, with an average of 19.2 kg/month and standard deviation of 0.88 kg/month. Apart from the prescription trends for the whole region variation across the tool helps to monitor individual API prescription by month (Fig. 3A), by individual GP level (Fig. 3B), by individual postcode level (Fig. 3C) and medicinal form for the selected CCG region (Fig. 3D) over the selected year.

The second dashboard in PrAnaViz generates spatiotemporal trends for an API. It helps to compare prescription quantity of an API by individual postcode level in a selected region over a period and it helps to generate annual trends. For example, as shown in Fig. 4A, prescription quantity of clarithromycin in January 2018 at an individual postcode levels in the Bath and North East Somerset CCG region, 77% of the practices in that postcodes prescribed more than 0.1 kg/month; while the top 17% in that prescribed more than 0.35 kg/month, For comparison, the prescription of more than 0.1 kg/month of clarithromycin at the practices in these postcodes for the month June 2018 decreased to 47%; while only one GP practice prescribed more than 0.35 kg/month in that period. Users can also visualise these results as a bar plot, as in Fig. 4C.

The tool also visualises the annual trends of clarithromycin at an individual postcode (for example, BA1 7NP in Fig. 4B) with an information about the corresponding GP practices at the postcode as presented in Fig. 4B.

## Discussion

### Summary

We have developed an R package, PrAna to calculate prescribed quantity (in kg) of an APIs by postcode using England’s national level prescription data provided by National Health Service, for the years 2015–2018. There were 27,848 unique BNF codes identified in the compiled prescription datasets, after excluding compounds related to medical devices/appliances, bath additives, washes, foams, shampoos, sprays, multivitamin capsules, and tablets, these were matched to 97% of the BNF codes generated. We have also created PrAnaViz, an in-built visualisation tool within the R package where user can explore spatiotemporal trends in prescription of an API or group of APIs. This tool helps to understand the GP practice level and postcode level variation of the prescribed drug in the selected CCG region, with resolution to each month and different medicinal forms. The tool also enables to download the generated dataset in comma separated value (.csv) files and publication ready images. Users can modify the colour palette for the navigation bar, side bar, accents bar and the plots colour schemes. The colour palette includes colour-blind/publication/printer friendly options.

### Strength and limitations

The R package enables to process annual data for the whole of England’s prescription data for the years 2015–2018, not a sample. The tool successfully calculated the quantity of APIs prescribed in several presentation levels such as tablets, capsules, oral form. We have used ‘Quantity’ from the NHS prescription dataset to measure total prescription quantity. The major limitations with the calculations are (1) the data used for the calculations are monthly time resolution, (2) delay in the release of the data, and (3) in some cases, people live far from their registered GP practices, for example, GP practices in the university premises, and (4) in some cases, the prescribed medicines were dispensed in a pharmacy located in a different city. We have developed PrAnaViz, a free, openly accessible, in-built interactive tool to visualise and analyse PrAna generated dataset in real-time. PrAnaViz facilitates wider use with spatiotemporal and long-term trends. The tool enables to calculate and visualise prescription quantity (in kg) per postcode, medicinal form, and GP practice. Users can download the produced graphs as a publication ready image or a dataset (.csv) to carry out their own analyses using different software.

We have installed a demo version of the PrAnaViz tool as web application with limited data, hosting only 2018, Bath and North east somerset CCG dataset and it is publicly available to get feedback and to monitor user volume. The tool is under continuous development.

## Conclusion

We have developed an R package, PrAna, incorporated with a method to aggregate and normalise NHS prescription dataset to calculate total prescription quantity for an individual API specified to a postcode or GP practice. We have also successfully calculated the total prescription quantity for England for the year 2015–2018. Apart from the R package, we have also developed a standalone, comprehensive, PrAnaViz, with the generated dataset to analyse, visualise, and explore prescription data. With its spatio-temporal prescribing trends of different APIs including resolution to postcode and GP Practices, PrAna and PrAnaViz, can be used as a support tool for policymakers, academics, and researchers in public healthcare, and by environmental scientists to monitor different group of pharmaceuticals emitted to the environment and for prospective risk assessment of pharmaceuticals in the environment.

## Data Availability

The source code, manuals and sample data are available for download from the GitHub repository https://github.com/PrAnaViz/PrAna. Availability and requirements: Project name: PrAna. Project home page: https://github.com/PrAnaViz/PrAna. Operating system(s): Platform independent. Programming language: R. Other requirements: R packages: shiny, leaflet, ggplot2, dplyr, lubridate, zoo, tidyr, reshape2. Licence: MIT License. Any restrictions to use by non-academics: N/A.

## Abbreviations

ADDR: General practice address files
AMP: Actual medicinal product
AMPP: Actual medicinal product pack
API: Active pharmaceutical ingredient
BANES: Bath and North East Somerset
BNF: British National Formulary
BSA: Business Services Authority
CCG: Clinical commissioning group
CRAN: Comprehensive R archive network
dm + d: Dictionary of medicines and devices
GP: General practice
MDR: Master data replacement
NHS: National health service
NSPL: National statistics postcode lookup
ONS: Office for national statistics
SNOMED: Systematized nomenclature of medicine
UK: United Kingdom
VMP: Virtual medicinal product
VMPP: Virtual medicinal product pack
